# Accounting for misclassification bias of binary outcomes due to underscreening: a sensitivity analysis

**DOI:** 10.1186/s12874-017-0447-9

**Published:** 2017-12-12

**Authors:** Nanhua Zhang, Si Cheng, Lilliam Ambroggio, Todd A. Florin, Maurizio Macaluso

**Affiliations:** 1 Division of Biostatistics and Epidemiology, Cincinnati Children’s Hospital Medical Center, 3333 Burnet Ave, MLC 5041, Cincinnati, OH 45229 USA; 2 Department of Pediatrics, University of Cincinnati College of Medicine, Cincinnati, OH USA; 3 Division of Emergency Medicine, Cincinnati Children’s Hospital Medical Center, Cincinnati, OH USA

**Keywords:** Misclassification, Selection model, Underscreening, Radiographic pneumonia

## Abstract

**Background:**

Diagnostic tests are performed in a subset of the population who are at higher risk, resulting in undiagnosed cases among those who do not receive the test. This poses a challenge for estimating the prevalence of the disease in the study population, and also for studying the risk factors for the disease.

**Methods:**

We formulate this problem as a missing data problem because the disease status is unknown for those who do not receive the test. We propose a Bayesian selection model which models the joint distribution of the disease outcome and whether testing was received. The sensitivity analysis allows us to assess how the association of the risk factors with the disease outcome as well as the disease prevalence change with the sensitivity parameter.

**Results:**

We illustrated our model using a retrospective cohort study of children with asthma exacerbation that were evaluated for pneumonia in the emergency department. Our model found that female gender, having fever during ED or at triage, and having severe hypoxia are significantly associated with having radiographic pneumonia. In addition, simulation studies demonstrate that the Bayesian selection model works well even under circumstances when both the disease prevalence and the screening proportion is low.

**Conclusion:**

The Bayesian selection model is a viable tool to consider for estimating the disease prevalence and in studying risk factors of the disease, when only a subset of the target population receive the test.

## Background

It is often of interest to estimate the prevalence of a certain condition in a population and this proportion is underestimated as those who were not screened for the disease were assumed negative for the condition. Under-screening of a disease is common because of resource limitation, perceived low risk which eliminates the need of screening, or lack of recommendations from guidelines. For example, fatty liver disease and metabolic syndrome among children may go undetected because relevant evaluations were not routinely recommended by pediatricians [1]. At patient level, under-diagnosis could cause delay in treatment. A study in Italy reported the rate of Chronic Obstructive Pulmonary Disease (COPD) under-diagnosis ranges between 25 and 50%, and as a consequence, many patients missed the optimal time for therapeutic intervention, contributing to the progression of the disease to be more severe [2]. Other common diseases that go underdiagnosed include hepatitis C virus (HCV) [3, 4], HIV and sexually transmitted diseases (STD) [5], hypertension in children and adolescents [6], and depression [7]. Under-diagnosis of infectious disease such as pneumonia, HIV or STD poses a societal burden.

Perceived as at low risk for the condition, those who are not screened or examined are usually classified as negative, which results in an underestimated proportion or prevalence of the disease in the study population. This misclassification can also bias the association of risk factors with the disease condition [8–10]. Standard approach to handle misclassification in binary outcomes relies on validation study of a subsample of initial non-respondents in the study population. When validation data are not available, Hausman et al. (1998) [11] and Savoca (2011) [12] examine the misclassification bias as a function of the error rates, under balanced and unbalanced scenarios. However, these methods require assumptions on the functional form of the positive diagnosis probability and the misclassification parameters [12]. Shebl et al. (2012) develop a likelihood-based method to estimate incidence when disease status is measured imperfectly based on hidden Markov models, while assuming the known constant levels of sensitivity and specificity and constant incidence rates over time [4].

Technically, the disease condition for those who were not screened/examined is unknown and classifying them as negative is based on a strong assumption about the missing values. Instead, we formulate the problem as a missing data problem and treat the disease status of those not tested as missing. The missing data mechanism, which concerns how the data are missing and whether the missingness is related to the underlying missing values, is critical when dealing with missingness. When the missingness depends neither on observed nor missing values, the data are missing completely at random (MCAR). When the missingness depends on the observed values but not the missing values, the missing data mechanism is called missing at random (MAR). In the case when the missingness can depend on the missing values, the missing data mechanism is called missing not at random (MNAR). When the missing data mechanism is MCAR or MAR, correct inference may be achieved based on a likelihood function which does not involve a modeling for the missingness mechanism; likelihood inference which ignores the model for missingness (ignorable likelihood, Zhang and Little 2011 [13]) includes maximum likelihood estimation, Bayesian inference, and multiple imputation [14]. However, when the missing data mechanism is MNAR, a correct inference has to consider the joint distribution of the outcome variable and the missingness indicator; depending on factorization of the joint distribution of the outcome variable and the missingess indicator, three classes of models have been investigated: the selection model, pattern mixture model, and shared parameter model [15].

In this article, we propose a class of Bayesian selection model, which estimates the disease prevalence in the study population (both screened and unscreened) using a sensitivity parameter which denotes the likelihood of being screened. This model will yield estimates of the prevalence as well as the association of risk factors with the disease outcome under different values of the sensitivity parameter and therefore the big picture of the research questions.

## Methods

### Data source

We were interested in estimating the proportion of children with an asthma exacerbation who were diagnosed with radiographic pneumonia among those presented to the emergency department of the Cincinnati Children’s Hospital Medical Center between January 1st, 2010, and December 31, 2013. Children were identified using a validated algorithm of *an International Classification of Diseases, Ninth Revision, Clinical Modification* diagnosis code of asthma (code 493.x) in the first 3 diagnosis positions and receipt of 1 or more doses of albuterol sulfate in the emergency department [16]. Children less than 2 years were excluded to minimize including infants with bronchiolitis.

We investigated the risk factors for radiographic pneumonia i.e. focal opacity present on chest radiograph) [17]. Consequences of the overuse of radiography include increased time in the hospital, unnecessary radiation, increased cost, and inappropriate antibiotic use due to equivocal imaging findings [18]. Due to the high rate of normal chest radiograph and the consequences of unnecessary radiograph, only about a third of those who presented to emergency department received chest radiography. This was noted as a limitation of the regression analysis used as only subjects who received chest radiography were included in fitting the model that assessed risk factors for radiographic pneumonia and therefore limited the generalizability of their findings to the larger study population of all children with asthma exacerbation who present to the ED [17]. Due to the fact that those who received chest radiography are a biased sample of all presented to ED with asthma exacerbation, with possibly higher probability of having radiographic pneumonia than children who did not receive the chest radiography, the analyses that discarded the subjects who did not receive the chest radiography may have led to biased estimation of the risk factors with the outcome of radiographic pneumonia.

Assuming those who did not undergo chest radiography to be negative for radiographic pneumonia will underestimate the prevalence of the disease in the study population and potentially bias the association of risk factors on the disease. We formulate this problem as a missing problem and use a Bayesian selection model to jointly model the disease status and the response indicator, i.e., whether the subject received chest radiography or not.

### Bayesian selection model

Let *y*_i_ denote the actual binary radiographic pneumonia status, equal to 1 if the *i* ^th^ subject had radiographic pneumonia, and 0 if not. This outcome was observed for subjects who received chest radiography, and missing for subjects who did not receive chest radiography. We use *R*_i_ to denote whether we observed the *i* ^th^ subjects radiographic pneumonia status, and *R*_i_ is equal to 1 if the subject received chest radiography and equal to 0 if not. We use *x*_i_ and *z*_i_ to denote the covariate sets that predicts the outcome *y*_i_ and the response status *R*_i_, respectively. The covariates in *x*_i_ and *z*_i_ may overlap with each other. The selection model is based on the joint distribution of (*y*_i_, *R*_i_),

1 $$ f\left({y}_{\mathrm{i}},{R}_{\mathrm{i}},{x}_{\mathrm{i}},{z}_{\mathrm{i}},\beta, \lambda, \theta \right)=f\left({y}_{\mathrm{i}},{x}_{\mathrm{i}},\beta \right)f\left({R}_{\mathrm{i}},{y}_{\mathrm{i}},{z}_{\mathrm{i}},\lambda, \theta \right) $$

where *f*(*y*_i_| *x*_i_; *β*) and *f*(*R*_i_| *y*_i_, *z*_i_; *θ*) are modeled as logistic regression as

2 $$ \mathrm{logit}\left(\Pr \left({y}_{\mathrm{i}}=1|{x}_{\mathrm{i}};\beta \right)\right)={x}_i^T\beta $$

3 $$ \mathrm{logit}\left(\Pr \left({R}_{\mathrm{i}}=1,{y}_{\mathrm{i}},{z}_{\mathrm{i}},\theta \right)\right)={z}_i^T\theta +\lambda {y}_{\mathrm{i}} $$

Here the parameter *β*denotes the risk for radiographic pneumonia, which is the main parameter of interest; and the parameters *θ*and *λ* relate the propensity of receiving chest radiography (and hence the response indicator) to covariates *z*_i_ and the actual pneumonia status *y*_i_. Note here *y*_i_ is missing for subjects who did not receive radiography, which leads to identification issues for this joint model [15].

To address the identification issues inherent in the model, we use*λ*as a sensitivity parameter, taking a range of fixed values from - ∞ to∞. When*λ*is 0, the propensity of a subject receiving chest radiography does not depend on this subject’s radiographic pneumonia status; this corresponds to missing at random assumption in the missing data literature. When*λ*is greater than 0, the propensity of a subject receiving chest radiography is positively associated with the subject’s radiographic pneumonia status. When*λ* is less than 0, having radiographic pneumonia is associated with lower propensity of receiving a chest radiography. For this specific application, it is reasonable to assume that patients with radiographic pneumonia are more likely to receive chest radiography than patients without radiographic pneumonia, and therefore *λ*> 0.

An important feature of the model is that by allowing the sensitivity parameter*λ* to change, we can assess how the main parameters of interest is sensitive to the perturbation of the sensitivity parameter. The Bayesian modeling setup also makes it easy to predict the overall proportion of the radiographic pneumonia in the study population for a fixed*λ*. For illustration purpose, we use the same set of covariates for *x*_i_ and *z*_i_, which includes gender (female vs. male), age at visit (≥ 5 years vs. < 5 years), fever during ED stay or at triage (temperature ≥ 38 °C vs. < 38 °C), and severe hypoxia (Oxygen saturation < 90% vs. ≥ 90%). [17]

We formulate the model in a Bayesian framework (BSM) and estimate the parameters using Markov Chain Monte Carlo (MCMC) methods. The MCMC algorithm is called “data augmentation”. The algorithm iteratively draws the next values of parameters and the unobserved *y*_i_ ‘s from the corresponding posterior distributions of the parameters and the posterior predictive distributions of the unobserved *y*_i_ ‘s. We use proper and non-informative prior distributions for all parameters, i.e., multivariate normal priors with mean 0 and diagonal covariance matrices with a large scale parameter of 10,000 for both*β*and*θ*. The software package WinBUGS is used to estimate the posterior distribution of the parameters [19].

### Results

Out of the 14,007 children who visited emergency department for asthma exacerbation, chest radiography was performed on 4708 children (33.6%). Radiographic pneumonia was present in 280 of the 4708 children who received chest radiography (5.9%).

**Fig. 1:**
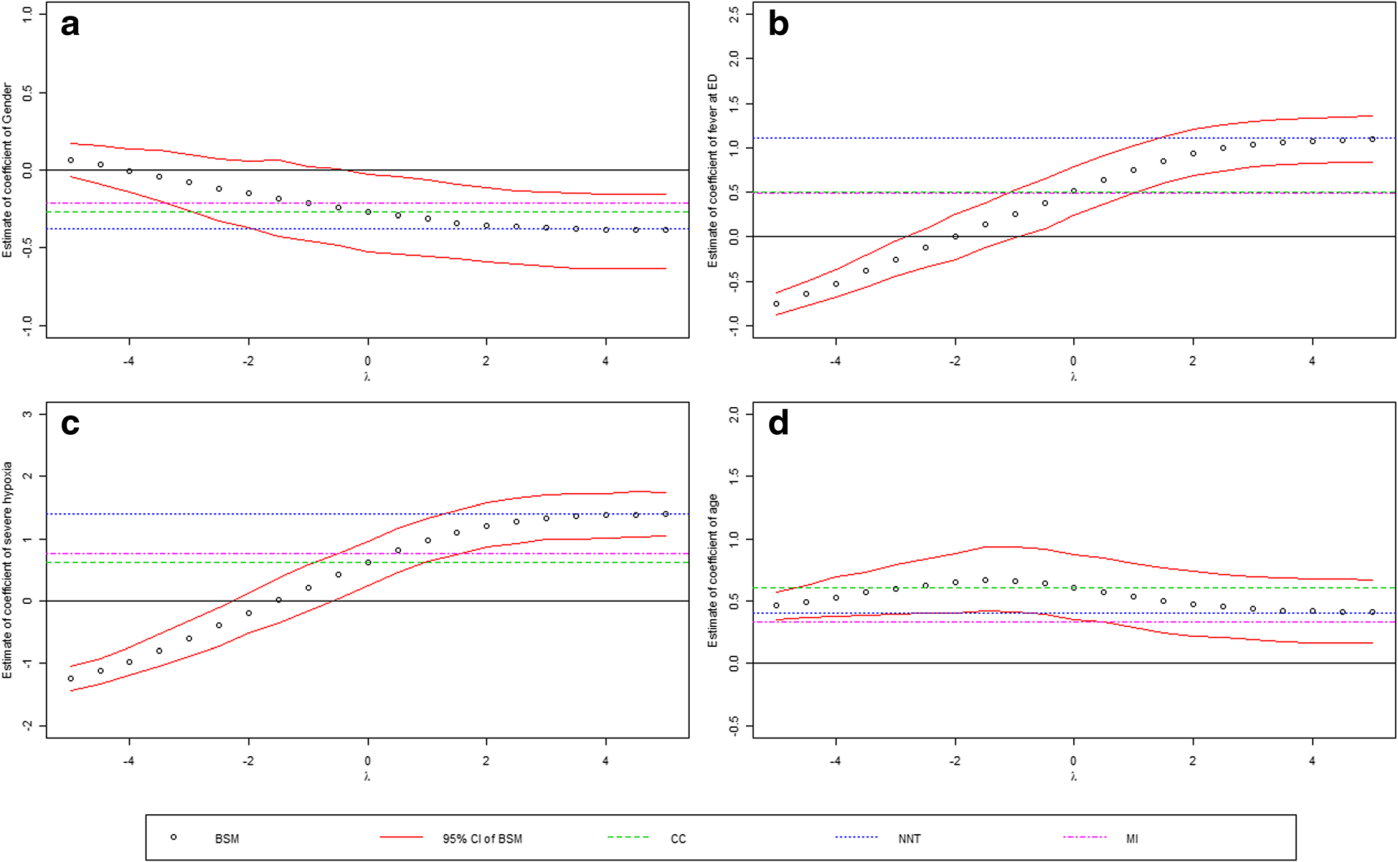
Regression coefficients of risk factors predicting radiographic pneumonia over sensitivity parameter λ. Figure 1(**a**), (**b**), (**c**), (d) shows the estimates of gender (male vs. female), fever at ED (yes vs. no), severe hypoxia (yes vs. no), and age. The results from the complete-case (CC) analysis, and the analysis which assumes negative for those not tested (NNT) are also shown

Figure 1(a)-(d) shows the regression parameters of gender (*β*_1_: males vs. female), age at visit (*β*_2_: ≥ 5 years vs. < 5 years), fever during ED stay or at triage (*β*_3_: temperature ≥ 38 °C vs. < 38 °C), and severe hypoxia (*β*_4_: Oxygen saturation < 90% vs. ≥ 90%), respectively. For comparison purpose, the results from the following two naïve methods were also plotted on the same plots:

- Complete-case analysis (CC): logistic regression only includes those had observed radiographic pneumonia status, i.e., those who received chest radiography;
- Negative for not tested (NNT): logistic regression with all subjects which assumes negative radiographic pneumonia for those who did not receive chest radiography.
- Multiple imputation (MI): multiple imputation using chained equation which assumes missing at random.

The point estimates from the four methods and the 95% credible intervals (CI) from the proposed Bayesian selection model were plotted on the same plots. As the sensitivity parameter goes from −4 to 4, we see a decreasing trend of the risk of having radiographic pneumonia comparing males to females. As we mentioned before, the true *λ* should be positive because those with pneumonia are believed to be more likely to receive chest radiography; therefore, we focus on the results when*λ*is positive. When*λ*is greater than 0, the coefficients is negative for gender from the BSM and the 95% CI does not cover 0, implying that males had significantly higher risk of having radiographic pneumonia than females among these who visited emergency department for asthma exacerbation (Fig. 1(a)). For*λ*> 0, old age is associated with significant high risk of having radiographic pneumonia among this study population (Fig. 1(b)). Having fever during ED or at triage, or having severe hypoxia, are both significantly associated with positive radiographic pneumonia (Fig. 1(c)-(d)).

For the estimates of these risk factors (Fig. 1), the Bayesian selection model yields the same results as the complete-case (CC) analysis when *λ*= 0; this is not surprising because when the missingness depends on the covariates but not the outcome, the complete-case analysis for fully efficient for the regression [13] When *λ*is sufficiently large (e.g., approaching to 4 in this example), the BSM methods yields results close to that of NNT. This is because when *λ*is large, it is sufficient to assume that those who did not receive chest radiography were negative for radiographic pneumonia. MI yields estimates close to CC for gender, fever at ED, severe hypoxia but smaller effect for age.

**Fig. 2:**
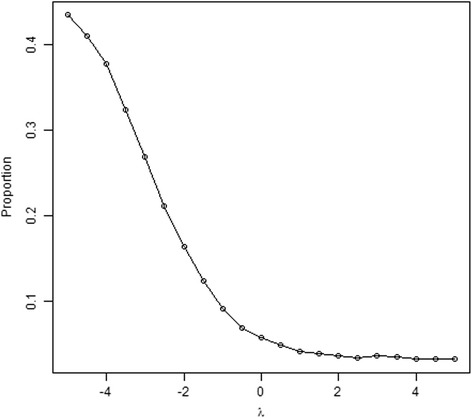
Estimated prevalence of radiographic pneumonia in the target population by sensitivity parameter λ

Figure 2 shows the overall prevalence of radiographic pneumonia in the study population decreases as sensitivity parameter*λ*increases. When *λ*is between 0 and 4, the estimates of the prevalence range from 0.056 to 0.032.

### Simulation studies

The prevalence of radiographic pneumonia in the current study population is less than 6%, which is relatively low. In some other diseases such as sexually transmitted diseases and hypertension, the prevalence could be much higher. Logistic models are well-known to suffer from bias for rare events, and therefore the prevalence has an impact on the proposed BSM method [20]. On the other hand, missingness proportion plays an important role in the performance of missing data models, and therefore in our setting the performance of the BSM method could also be affected by the proportion of screening. In this section, we assess the performance of the BSM for different values of disease prevalence and proportions of subjects screened.

We generate two covariates, *x*_1_ which is a binary variable from Bernoulli distribution (e.g., gender) and *x*_2_ from a normal distribution (e.g., age of high school students), and *b*_1_, *b*_2_ are the regression coefficients of *x*_1_, *x*_2_ in the main outcome model, respectively,

$$ {x}_1\sim \mathrm{Bernoulli}\ (0.5) $$

$$ {x}_2\sim N\left(16,2\right) $$

and we generate the disease status *y* based on a logistic regression model,

$$ \mathrm{logit}\left[\Pr \left(y=1\right)\right]=a+{x}_1+{x}_2 $$

and the response indicator *R* is also generated based on a logistic regression model,

$$ \mathrm{logit}\left[\Pr \left(R=1\right)\right]=b-{x}_1+{0.5}^{\ast }{x}_2+{0.5}^{\ast }{y}_1 $$

**Fig. 3:**
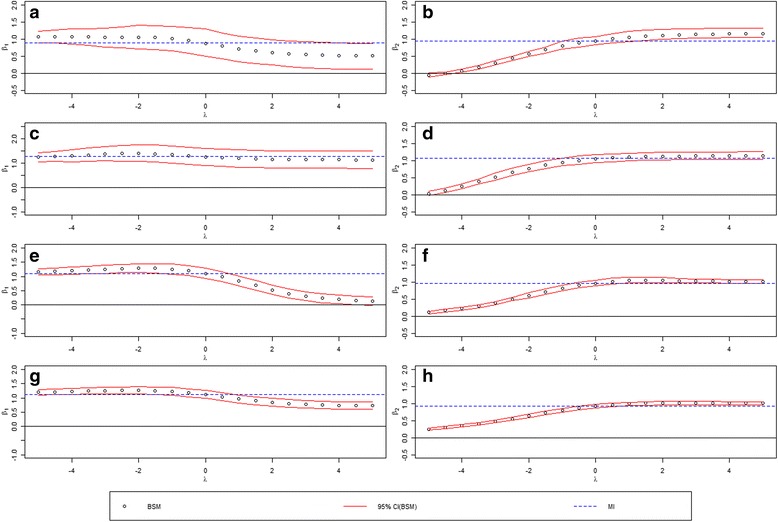
Regression coefficients of *x*_1_ and *x*_2_ under four different levels of prevalence and screening rates (Low-Low, Low-High, High-Low, High-High). Figure 3(**a**), (**b**) shows the regression coefficients of *x*_1_ and *x*_2_ under low-low scenario, Fig. 3(**c**), (**d**) shows the regression coefficients of *x*_1_ and *x*_2_ under low-high scenario, Fig. 3(**e**), (**f**) shows the regression coefficients of *x*_1_ and *x*_2_ under high-low scenario, and Fig. 3(**g**), (**h**) shows the regression coefficients of *x*_1_ and *x*_2_ under high-high scenario

**Fig. 4:**
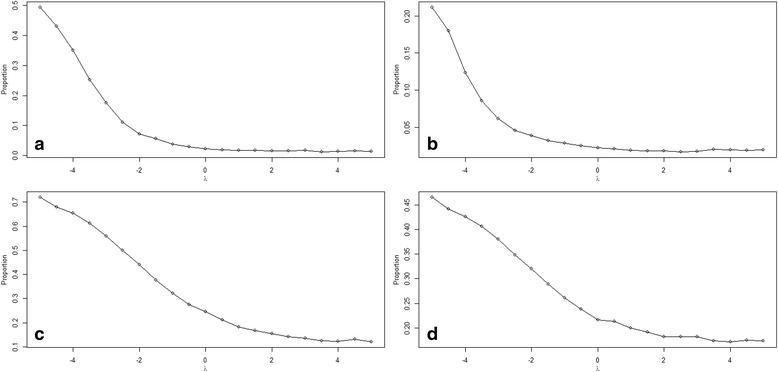
Estimated prevalence by sensitivity parameter λ under different levels of prevalence and screening rate: (**a**) Low-Low, (**b**) Low-High, (**c**), High-Low, and (**d**) High-High

We choose *a* to be −22.2 and −18.7 so the prevalence is around 2% (low prevalence) and 20% (high prevalence), and *b* to be −8.5 and −7.0 so that the response rates are 30% (low screening rate) and 60% (high screening rate). The combinations result in four simulation scenarios: (1) low prevalence and low screening rate; (2) low prevalence and high screening rate; (3) high prevalence and low screening rate; (4) high prevalence and high screening rate. We simulate 10,000 subjects from the study population and plot the regression estimates vs. the sensitivity parameter for each regression coefficient in Fig. 3, and the estimated prevalence vs. the sensitivity parameter in Fig. 4. To assess the performance of the BSM methods under the true sensitivity parameter (λ = 0.5), we replicate the process 200 times and evaluate the methods by assessing the empirical bias, the root mean squared error, and the coverage probabilities of the 95% credible interval.

Table 1 shows the bias, RMSE and coverage probability of the 95% credible intervals of the proposed method along with NNT, CC and MI methods, when the sensitivity parameter is set at the true value (0.5). The true values of the regression coefficients of *x*_1_ and *x*_2_ are both 1, and the empirical bias is less than 1% for all but one coefficient, out of all simulation scenarios for the BSM method; Only the coefficient of *x*_2_ shows more than 1% empirical bias under the scenario when both prevalence and the screening proportion are low. We see an improvement in the RMSEs (i.e., smaller RMSEs) with increase in either the disease prevalence or the screening proportion. As expected, the coefficient of the binary covariate *x*_1_ has larger RMSEs than the coefficient of the continuous covariates *x*_2_. In general, the BSM method achieves good coverages for both regression coefficients. The two cases with coverage probabilities less than 90% are for the coefficient of *x*_2_ when the screening probability is low. All other methods show large bias, increased RMSE and poor confidence coverage compared to BSM method.

**Table 1:** Bias, RMSE, and Coverage Probability of 95% Credible Interval of the BSM, NNT, CC, and MI method, When Sensitivity Parameter is Set to True Value

			Low-Low	Low-High	High-Low	High-High
BSM	Bias*1000	*b*_1_	3.19	4.14	1.91	2.88
		*b*_2_	12.94	4.81	2.60	1.88
	RMSE*1000	*b*_1_	192.51	163.04	94.14	66.59
		*b*_2_	75.19	54.64	40.47	28.12
	Coverage Probability	*b*_1_	96.0%	97.0%	97.0%	97.5%
		*b*_2_	88.0%	94.5%	88.5%	91.0%
NNT	Bias*1000	*b*_1_	−309.37	−101.24	−857.09	−331.60
		*b*_2_	136.93	56.88	30.70	34.85
	RMSE*1000	*b*_1_	362.4	190.85	860.28	338.08
		*b*_2_	151.02	77.23	41.93	42.98
	Coverage Probability	*b*_1_	63.0%	95.0%	0%	0%
		*b*_2_	43.5%	86.0%	81.0%	73.5%
CC	Bias*1000	*b*_1_	89.30	43.30	113.73	77.02
		*b*_2_	−38.4	−24.67	−51.17	−37.12
	RMSE*1000	*b*_1_	212.04	167.71	148.09	101.61
		*b*_2_	81.55	60.70	64.04	45.83
	Coverage Probability	*b*_1_	93.0%	96.0%	76.5%	83.5%
		*b*_2_	89.5%	91.5%	68.5%	74.5%
MI	Bias*1000	*b*_1_	97.01	43.23	115.96	79.41
		*b*_2_	−43.32	−25.72	−51.34	−35.10
	RMSE*1000	*b*_1_	221.17	168.02	150.53	103.85
		*b*_2_	84.14	61.07	64.81	44.29
	Coverage Probability	*b*_1_	92.5%	97.0%	76.0%	82.0%
		*b*_2_	88.0%	92.5%	71.5%	76.0%

*denotes "multiplied by"

*BSM* Bayesian selection model, *NNT* Negative for not tested, *CC* Complete-case analysis, *MI* Multiple imputation

Figure 3 shows the point estimates plots along with the 95% credible intervals of the two regression coefficients of *x*_1_ and *x*_2_, under the four simulation scenarios. Figure 3(a), (c), (e) and (g) are for coefficients of *x*_1_ under (1) low prevalence and low screening rate, (2) low prevalence and high screening rate, (3) high prevalence and low screening rate, (4) high prevalence and high screening rate, respectively, while Fig. 3(b), (d), (f), (h) are for coefficient of *x*_2_ under the corresponding four simulation scenarios. We see similar trends in the coefficient estimates with the change of the sensitivity parameter. However, there is substantial improvement in precision (tighter confidence bands) with the increase of prevalence or screening proportion. The estimated prevalence under different sensitivity parameters for the four simulation scenarios were plotted in Fig. 4.

## Discussion

Under-screening results in missing disease status or misclassified disease status when assumptions are made for those who did not receive the screening test. The goal of our method was to demonstrate the use of the Bayesian selection model for missing outcome or misclassified outcome due to under-screening. Unlike other methods that rely on assumptions [8, 9] or validation data, [12] the BSM method relates the propensity of receiving screening to the disease status through a sensitivity parameter. By varying the sensitivity parameter, the BSM method demonstrated how the prevalence and the association of risk factors change with the sensitivity parameter. We further used simulation studies to demonstrate the performance of BSM method under different levels of disease prevalence and screening proportion. Our simulation indicates that the BSM method performs well even under scenarios when both the prevalence and the screen proportion are low.

For illustration purpose, we applied the proposed BSM method to a pneumonia dataset. The results showed increased risk of pneumonia in girls, which is consistent with studies from Japan [21]. The results also indicated that having fever during ED or at triage, or having severe hypoxia, is positively associated with radiographic pneumonia. This is not surprising, as both fever and hypoxia are symptoms of pneumonia in kids [22]. A more rigorous analysis of the risk factors for radiographic pneumonia would need to examine more risk factors and possibly their interactions.

The Bayesian selection model is an important tool to consider for estimating the disease prevalence and in studying risk factors of the disease, when only a subset of the target population receive the test. For studying the association of the risk factors, i.e., the regression of outcome on risk factors, this method reduces to the complete-case analysis when the sensitivity parameter is set to zero, and approximates the NNT method when the sensitivity parameter approaches infinity. Unfortunately, there is no information available to estimate the sensitivity parameter without validation sample. The choice of the sensitivity parameter can be aided by gathering information relating the propensity of receiving the test to the actual disease status. The choice of covariates in the outcome model and the response indicator model can be aided by input from substantive experts regarding the hypothesized relationship of variables with the outcome and/or the response indicator. When validation data are available, it is possible to identify the parameters in the Bayesian selection model. In future work, we plan to study how to efficiently make use of the validation data.

## Conclusions

In the current study, we developed a Bayesian selection model that jointly modeled the binary outcome and the response indicator for the case when the binary outcome may be missing or misclassified due to under-screening. The model for the response indicator relates the propensity of receiving screening to the disease status through a sensitivity parameter. The application of the model to a pneumonia data yielded results that were consistent with previous studies. The performance of the proposed method over other methods in the simulation studies demonstrated the promise of the proposed model for modeling missing or misclassified disease outcome due to under-screening.

## Abbreviations

BSM: Bayesian selection model
CC: Complete-case analysis
CI: Confidence interval
COPD: Chronic obstructive pulmonary disease
ED: Emergency department
HCV: Hepatitis C virus
MAR: Missing at random
MCAR: Missing completely at random
MCMC: Markov chain Monte Carlo
MI: Multiple imputation
MNAR: Missing not at random
NNT: Negative for not tested
RMSE: Root mean squared error
STD: sexually transmitted disease
